# Deep Convolutional Neural Networks for Autofocus Control on a *C. elegans* Tracking System

**DOI:** 10.3390/bios16020119

**Published:** 2026-02-12

**Authors:** Santiago Escobar-Benavides, Jose-Julio Peñaranda-Jara, Joan-Carles Puchalt, Antonio-José Sánchez-Salmerón

**Affiliations:** Instituto de Automática e Informática Industrial, Universitat Politècnica de València, Camino de Vera S/N, 46022 Valencia, Spain; sanesbe1@etsii.upv.es (S.E.-B.); jjpeajar@epsg.upv.es (J.-J.P.-J.); juapucro@doctor.upv.es (J.-C.P.)

**Keywords:** convolutional neural networks, autofocus, deep learning, supervised learning, *C. elegans*

## Abstract

Correct focal positioning is essential for microscopy imaging of live moving subjects such as *Caenorhabditis elegans*. However, many methods can be too slow to perform real-time control to keep the subject in focus. In this work, we propose a convolutional neural network-based method to perform one-shot prediction of the optimal focusing distance, without the need to scan iteratively the optical axis to find the optimal position. A new data augmentation technique is proposed, and its effectiveness is validated through statistical analysis. This technique is shown to improve results without the need for additional data collection. Several architectures are trained in z-stacks of images, using the proposed data augmentation technique, and compared on a validation set. Through this comparison, we find that the ConvNext V2, a novel architecture in this context, outperforms other models proposed in previous works. Furthermore, the impact of the Field of View used for the model’s prediction is studied, with the aim of further understanding the influence of spatial resolution and spatial compression on the performance of the model.

## 1. Introduction

The nematode *Caenorhabditis elegans* (*C. elegans*) has become a widely used model for biomedical research. Due to its size, of approximately 1 mm, its short lifespan, of approximately 3 weeks, and its ease of cultivation, the *C. elegans* is ideal for studying aging and neurodegenerative diseases, as well as for the screening of new drugs.

Despite their ease of manipulation, conducting assays with *C. elegans* has a high time cost, as laboratory technicians must spend a high number of hours observing and measuring each worm. For this reason, in recent years, there has been a great interest in the automation of these assays, with healthspan [1,2] and lifespan [3,4] being some of the most important examples.

Among the hardware solutions proposed for the automation of *C. elegans* imaging, devices such as [5,6,7] can be found, where some type of motorized XYZ stage enables the tracking of the individual *C. elegans*. This type of device offers the possibility of acquiring high-resolution images of individual worms, as the XY axis allows it to center the worm under the camera and the Z axis allows it to keep the worm in focus.

In this work we present a solution based on convolutional neural networks (CNNs) to allow for the automatic control of the Z axis in such types of devices. Traditionally, autofocus algorithms like [6,8,9,10] rely on searching along the focal axis to determine the optimal position that maximizes a given focus measure index. Given that for this application, the subject will be moving and therefore changing the section of the plate captured in the image, it is important to maintain continuous control and run in real time while the tracking is taking place. To address these challenges, we opted for a deep learning-based approach to enable rapid and adaptive focus adjustments without the need for exhaustive focal axis search.

Furthermore, the problem discussed is not limited solely to working with *C. elegans*, but rather the ability to perform real-time autofocus is beneficial in other micro-inspection use-cases, where the sample geometry, focal plane, or imaging context evolves during acquisition. In live-cell imaging of motile cells, cell migration, substrate interactions, and shape changes frequently induce axial motion or alter the effective focal plane. Similarly, in long-term developmental imaging—such as embryogenesis or organoid growth—gradual but continuous changes in sample thickness, volume, or position, as well as mechanical drift over multi-hour or multi-day experiments, can lead to progressive defocus if focus is not actively corrected during imaging. In surface inspection of non-planar or drifting samples, including micro-fabricated or tilted structures, variations in surface height and scanning-induced geometry changes result in spatially and temporally varying focus requirements, particularly at high magnification with limited depth of field. Across these applications, real-time autofocus enables sustained high-quality imaging without interrupting acquisition, rescanning regions of interest, or relying on repeated volumetric imaging, thus preserving temporal continuity, reducing data redundancy, and supporting downstream analysis that depends on consistent focus over time.

Similar approaches, for other applications, have been proposed in previous work. In [11] Xiang et al. show the feasibility of combining CNN with recurrent neural networks. In other works, deep learning models are combined with specific hardware setups. Ref. [12] Pinkard et al. show a method based on off-axis led illumination and [13] Ho et al. combine the use of phase detectors with a CNN architecture. In this work, we propose a CNN-based approach for estimating the focusing distance in a single-shot inference setting. While inspired by prior CNN-based autofocus methods [14,15,16], our contributions are as follows: (i) we introduce a novel data augmentation technique tailored for autofocus tasks, (ii) we apply deep learning-based autofocus to *C. elegans* imaging for the first time and (iii) we train and compare several architectures, identifying one not previously explored for autofocus that achieves state-of-the-art results.

Despite these advances, existing deep learning-based autofocus approaches exhibit several limitations that restrict their applicability in some microscopy settings, such as ours. Many prior methods rely on specialized optical configurations or additional hardware components, such as engineered illumination patterns or phase detection mechanisms, which increase system complexity and limit portability across microscope platforms. Other approaches focus primarily on architectural feasibility rather than comparative performance, often evaluating a single model without systematically assessing alternative network designs or establishing clear performance gains over existing CNN-based methods. These gaps highlight the need for autofocus solutions that achieve state-of-the-art accuracy using standard imaging hardware, operate in a single-shot inference regime, and are validated through rigorous architectural comparison. Our work directly addresses these limitations by demonstrating that improved autofocus performance can be achieved through architecture selection and task-specific data augmentation alone, without requiring any modification to the optical setup.

Moreover, as this novel data augmentation technique relies on the Field of View vs spatial compression relation, we conducted further experiments to determine the impact of the Field of View used in the input image on the model’s prediction.

## 2. Materials and Methods

### 2.1. Dataset

Adult *C. elegans* from strain N2 were used for data acquisition. The *C. elegans* were maintained at 20 °C and cultured on 55 mm Nematode Growth Medium (NGM) plates. *Escherichia coli* strain OP50 was used as standard prey for *C. elegans* in the laboratory.

The image capture system used consists of a Raspberry Pi HQ camera using a CS/C-mount lens with adjustable optical zoom (0.75x–5x) and a working distance of 90–97 mm. The camera is mounted on a 5 mm diameter trapezoidal lead screw with a 1 mm pitch, driven by a NEMA 17 stepper motor configured for 200 steps per revolution, allowing precise control of the lens movement along the focal axis.

To create the dataset required for training, we captured 74 stacks of images, each obtained by moving the Z-axis stepper motor one step at a time. This ensured that the same *C. elegans* specimen appeared in both defocusing directions. For ease of use and implementation, the images have been labeled with the number of steps to the optimal position.

For each stack, the Vollath F5 [17] index was computed for every image. This index gives a statistical focus measure of the image sharpness by analyzing spatial correlations between neighboring pixel intensities, capturing the degree of local structure in the image. We have chosen this index due to its simplicity, computational efficiency and robustness to noise. The Vollath F5 index is given by

(1) $$F_{\mathrm{voll}5}=\sum_{i=1}^{M-1}\sum_{j=1}^{N}g\left(i,j\right)\cdot g\left(i+1,j\right)-{\bar{g}}^{2}\cdot N\cdot M$$

where g(i,j) would be the grayscale intensity value of the image at pixel position (i, j), *M* the number of rows of the image, *N* the number of columns of the image and $\bar{g}$ the mean intensity value of the entire image.

**Figure 1:**
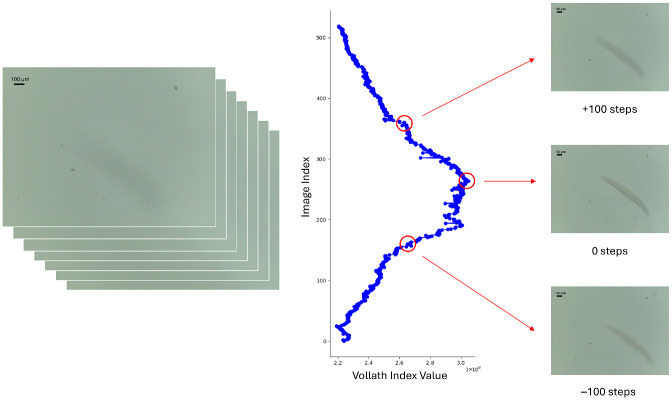
Example of labeled stack of images using Vollath F5 index.

The image with the maximum value for that index was selected as the best focused image. Every other image in the stack was labeled incrementally from that point, each image adding one step in each defocusing direction. An example of this labeling can be seen in Figure 1.

**Figure 2:**
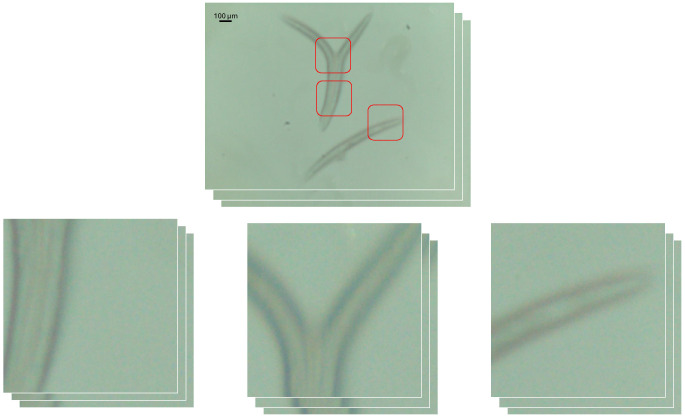
Example of cropped stacks from a complete stack. The red boxes indicate the crops shown below.

Due to real-time control requirements for the CNN, the input size of the image to the model will be limited to 500 × 500 pixels. This size was empirically chosen to ensure a good trade-off between processing speed and the amount of information still present in the image. As the original size of the images is 2592 × 1944 pixels, the images will suffer 4-5x spatial compression. Given that the most relevant focusing information can be found in the borders of the *C. elegans*, we extracted 500 × 500 pixel windows centered on the worms, creating more stacks that, in this case, will not suffer spatial compression, as can be seen in Figure 2. These stacks have been centered around the different *C. elegans* bodies, manually determining the center of each one of the cropping areas. These stacks have been labeled with the same method using the Vollath F5 index.

The use of these cropped windows could serve as a data augmentation technique during training or as a way of achieving higher precision during inference, as the cropping could be automated with the detection model needed to move the XY stage, such as the ones proposed for *C. elegans* detection in [18,19,20]. A detailed analysis of the impact of this technique follows in subsequent sections.

A total of 262 stacks were cropped from the original stacks. After this, the stacks have been split for training and validation. It should be noted that all of the *C. elegans* used for validation were captured from different Petri dishes to prevent any potential information leakage between the training and validation sets. A summary of the dataset split can be seen in Table 1.

### 2.2. CNN Architectures

We have trained multiple architectures to test which one could achieve a better performance on the validation set. As the positions along the focal axis are treated as a continuous variable, the models are formulated as regression models, directly estimating the focal distance from a single input image. As mentioned previously, the real-time control requirement greatly limits the size of the model that can be used; it is for this reason that we have not been able to select big CNN models that are not able to offer a good inference speed. We have trained and tested ResNet18 [21], closely matching the model presented in [16], two sizes of the MobileNetV3 architecture [22], as proposed in [15] and also two different sizes of the ConvNext V2 architecture [23]. The ConvNeXt V2 model was selected due to its strong performance in recent literature, where it has been widely adopted as a competitive CNN architecture. ResNet18 and MobileNetV3 were included because they have been employed in prior relevant studies. This selection enables a systematic comparison of different network architectures, allowing us to assess the impact of architecture choice on autofocus performance. A comparison between the models can be seen in Table 2, where some of the most important features are shown.

The hardware used to train the model includes a Ryzen 9 3900X processor with 12 cores running at 3.8 GHz, 128 GB of DDR4 3200 MHz memory (Manufacturer: AMD, Santa Clara, CA, USA), and a Nvidia RTX 3090 GPU with 24 GB of DDR4 memory (Manufacturer: Nvidia, Santa Clara, CA, USA).

All software used for data acquisition, processing, model training, and evaluation was developed in Python (3.8.20), using open-source libraries such as PyTorch (1.7.0), OpenCV (4.13.0.90) or Numpy (1.24.3).

## 3. Results

### 3.1. Training Results

To train all the models we used Mean Squared Error (MSE) as the loss function and the Adam optimizer with a learning rate of 0.0001. A learning rate scheduler (StepLR) was applied, reducing the learning rate by a factor of 0.85 every 5 epochs to ensure stable convergence. The models were trained for a total of 100 epochs. The batch size varied between 4 and 32, depending on the model size, and was always maximized based on the available GPU memory to optimize training efficiency.

For data augmentation, on the training set we have used random rotations up to 45°, random vertical flips and random horizontal flips.

For the evaluation of the performance of the models, we calculated the mean absolute error (MAE) of the validation dataset. In Table 3 the MAE is shown, with the mean values calculated separately for complete images and cropped images.

**Figure 3:**
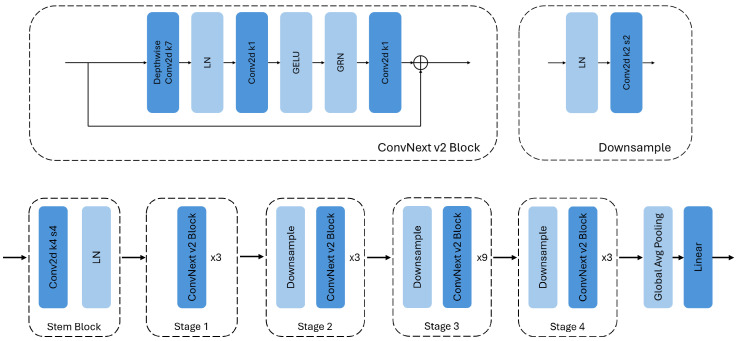
ConvNext V2 Tiny architecture diagram.

**Figure 4:**
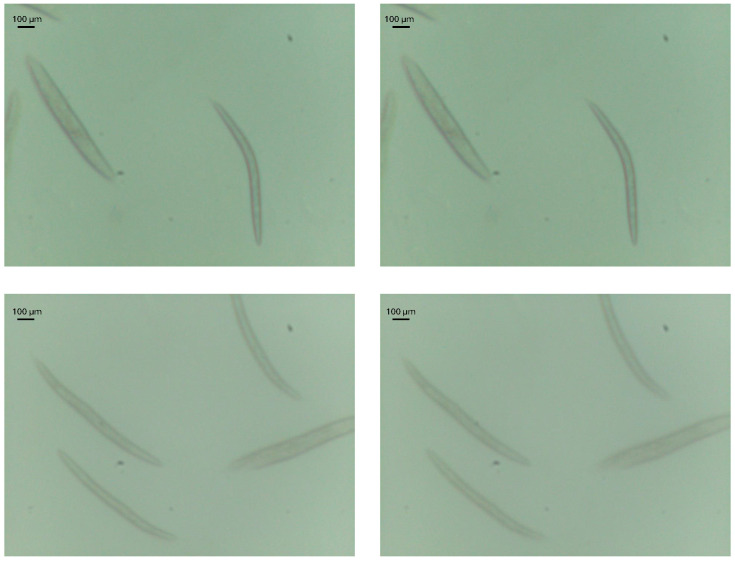
Comparison of *C. elegans* images at optimal focus (**left** column) and with a 25-step displacement along the Z axis (**right** column).

We can see that the ConvNext V2 model shows the best results after training, with the tiny version having the best performance across all models, achieving a MAE of 25 steps for the complete images. A diagram showing the architecture of the model ConvNext V2 Tiny in detail can be seen in Figure 3. For reference, given that with our setup, one step of the motor corresponds to 5 μm of lens displacement, and that the thickness of a *C. elegans* can be up to about 80 μm [24], this thickness would correspond to approximately 16 steps. This thickness of the *C. elegans* represents the axial range in which an idealized differential focal plane would intersect the body of the worm. In practice, the imaging system has a finite depth of field, so multiple Z positions, not only these 16 ideal positions, around the nominal optimum still yield images that are effectively well focused. This can be visually observed in Figure 4, where an image displaced by 25 steps from the optimal focal position retains sharp structural details and appears comparable to the optimal-focus image.

It can also be seen how, despite suffering a higher spatial compression, the ConvNext V2 tiny model is able to obtain better results on the complete images, showing worse performance on the cropped stacks.

### 3.2. Cropped Stacks Data Augmentation

To be able to determine whether adding the cropped images has a significant effect on the training, a ConvNext V2 tiny model has been trained on the complete images only, without including the cropped stacks. This model has been trained with the exact same hyperparameters previously mentioned. In Table 4 the evaluation of this model is compared with the previous training, in which the cropped stacks were included during training.

Furthermore, a statistical test has been carried out to determine whether the improvement in the results shown in Table 4 is statistically significant. Given that we are in a case where we are going to compare these models on the same validation dataset, we therefore have two paired sequences of errors. Given this, we have calculated the sequence of differences between the errors, given by

(2) $$d_{i}=e_{i}^{cropped}-e_{i}^{nocropped}$$

where $d_{i}$ is the difference between errors for image *i*, $e_{i}^{cropped}$ is the error of the model trained with the cropped stacks for image *i* and $e_{i}^{nocropped}$ is the error of the model trained without the cropped stacks for image *i*.

With this sequence of data points, we first checked if it follows a normal distribution, using the Shapiro–Wilk test. As we have obtained a *p*-value < 0.001, we can reject the null hypothesis of normality.

Then we applied the Wilcoxon signed-rank test to determine if there is a significant difference between the errors of both models [25], that is, to test whether the distribution of the differences in errors is symmetric around zero or not. As we obtained a *p*-value < 0.001, we can reject the null hypothesis that the median of differences is equal to zero and therefore affirm that the difference in using the proposed data augmentation technique is statistically significant.

### 3.3. Field of View Effect

Table 3 shows how the best-performing model, the ConvNext V2 tiny model, has a noticeable difference in performance between the complete images and the cropped images, which for us is a counter-intuitive result, since in the complete images a significant spatial compression is taking place on the focusing information present in the neighboring pixels of the worm edges. Because of this, we have performed a final experiment, seeking to understand the relationship between the Field of View present in the image and the model’s performance during inference.

**Figure 5:**
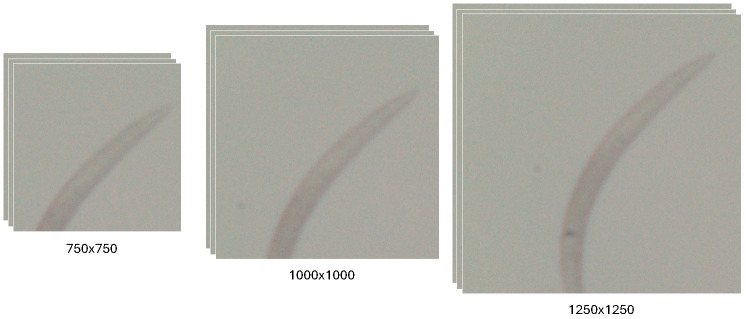
Example of cropped stacks with different sizes, 750 × 750, 1000 × 1000 and 1250 × 1250 pixels, respectively.

For this experiment, new stacks of cropped images have been created with different intermediate dimensions between 500 × 500 pixels and the complete image. As can be seen in Figure 5, stacks of 750 × 750, 1000 × 1000 and 1250 × 1250 pixels have been created, with 2128 images for each dimension.

With these stacks, inference has been performed with the ConvNext V2 tiny model and the mean absolute error has been calculated for each dimension. Table 5 shows these results, comparing them with the results obtained for the cropped images of 500 × 500 pixels and the full images.

## 4. Discussion

This paper has presented a method based on one-shot inference with convolutional neural networks to solve the autofocusing problem on *C. elegans* tracking assays. The best result achieved a mean absolute error of 25.849 steps in the validation dataset, with the thickness of a worm being up to 16 steps; thus, it is able to focus accurately on each nematode.

Different architectures were tested, where models already proposed in previous works for similar autofocusing tasks were compared against an architecture that had not been tried before, the ConvNext V2. This architecture, in its tiny version, achieved the lowest mean absolute error, surpassing the previously proposed architectures in about 47 and 67 steps respectively.

Furthermore, the results of Table 3 suggest that the potential of a model to achieve good results is heavily architecture-dependent, without necessarily showing a linear relationship with the number of parameters when comparing different architectures.

On the one hand, it can be seen how with the MobileNetV3 architecture, despite presenting the worst results and therefore the greatest potential for improvement, the large version of this architecture has only been able to match, and not surpass, the results of the small version, which has only half as many parameters.

On the other hand, it can be seen how the ConvNext V2 Pico model manages not only to equal, but also to significantly improve the results of the ResNet18 model, having approximately 25% fewer parameters.

In addition, a new method of data augmentation has been proposed, consisting of the use of stacks of cropped images, obtained from the complete images. This method is based on seeking to present the most relevant focusing information to the models without undergoing any spatial compression due to resizing. We have found that with this method, by combining complete stacks and cropped stacks during training, better performance is obtained. A statistical analysis has also been carried out, allowing us to confirm that the improvement due to this data augmentation technique is statistically significant.

Since it has also been observed that the ConvNext V2 tiny model, which obtains the best results, is able to obtain much better results on complete images than on cropped images, a study has been carried out to determine how the Field of View included in the image affects the performance of the model. For this, stacks of intermediate dimensions, of 750 × 750, 1000 × 1000 and 1250 × 1250 pixels, have been created to evaluate the model. Through this evaluation, it has been observed that, despite not having used these sizes during training, the tendency seen with the 500 × 500 cropped stacks is maintained.

The mean absolute error is smaller in these intermediate sizes with respect to the original crop size but larger than in the complete images, and within these intermediate sizes the best performance is observed in the 1250 × 1250 stacks. Despite this, the difference between the 750 × 750 and 1000 × 1000 stacks is only 0.6 steps, with no improvement in performance between these two sizes.

Although these results maintain the initial tendency observed, it is important to note that this trend was only observed in the model with the best results, while the remaining models showed similar performance between the complete and cropped images. We believe that this may be due to the fact that these models do not reach the point where they can exploit the difference between the two types of images; that is, they reach the point where the potential of the cropped images is saturated, but do not learn to extract further information from the complete images. However, the lack of interpretability of deep learning models means that these are merely assumptions.

Thus, from this study, we can conclude that although focusing information is primarily local, often concentrated near edges and neighboring pixels, better performance can be achieved by resizing images to capture a larger Field of View, even if it introduces some spatial compression. In other words, while high resolution preserves local details, it may be more beneficial to prioritize broader spatial context over perfect retention of fine-grained information.

Importantly, the observations discussed above are not specific to *C. elegans* imaging, but instead reflect more general properties of focus information in microscopy. As shown in our results, preserving broader spatial context can improve focus estimation even when fine-grained details are partially compressed, suggesting that the proposed approach is not tied to *C. elegans*-specific features but to more general image formation characteristics. This means that the methods proposed in this work could align and generalize to imaging conditions encountered in live-cell microscopy, long-term developmental studies, and micro-inspection of non-planar samples, where both local contrast variations and global structural patterns evolve continuously over time.

Furthermore, because the proposed method operates in a single-shot inference setting and does not rely on specialized optical configurations or hardware modifications, it could easily be integrated into a wide range of existing microscopy systems. This makes it particularly suitable for applications that require continuous, real-time imaging, such as tracking motile cells, monitoring slow developmental processes, or scanning extended surfaces with spatially varying focus. In these contexts, the ability to maintain accurate focus estimation without interrupting acquisition or increasing system complexity is critical. Taken together, these considerations suggest that the approach presented in this work proposes a solution that could generalize beyond *C. elegans* imaging to other dynamic micro-inspection scenarios that share similar real-time and robustness requirements.

## Figures and Tables

**Table 1:** Dataset split summary.

	Train		Validation	
	Stacks	Images	Stacks	Images
Complete Image Stacks	68	27,646	6	1988
Cropped Image Stacks	244	98,038	18	6528

**Table 2:** Comparison of total parameters (in millions), inference time for a 500×500 pixel image (in ms), and FLOPs (in GFLOPs) of each model used.

Model	Params (M)	GPU_time_ (ms)	CPU_time_ (ms)	GFLOPs
ResNet18	11.31	1.92	55.80	46.59
MobileNetV3 Small	2.82	3.74	86.88	0.33
MobileNetV3 Large	5.77	4.40	215.67	1.21
ConvNeXt V2 Pico	8.68	3.27	164.43	6.65
ConvNeXt V2 Tiny	28.05	7.39	601.35	21.67

**Table 3:** Mean absolute error (MAE) in steps of each model on the validation set, differentiating between complete and cropped images. The best result is shown in bold.

Model	Validation MAE_complete_	Validation MAE_cropped_
ResNet18	72.531	74.297
MobileNetV3 Small	92.765	105.767
MobileNetV3 Large	98.247	110.742
ConvNeXt V2 Pico	50.478	49.681
ConvNeXt V2 Tiny	**25.849**	**42.326**

**Table 4:** Comparison of training a ConvNeXt V2 Tiny model with and without the cropped stacks during training. MAE on complete and cropped validation stacks. The best results are shown in bold.

Training Dataset	Val. MAE_complete_	Val. MAE_cropped_
Complete stacks	40.595	100.252
Complete & Cropped stacks	**25.849**	**42.326**

**Table 5:** Mean absolute error (MAE) in steps for different cropping dimensions. The best result is shown in bold.

Cropping Dimension	MAE
500×500 pixels	42.326
750×750 pixels	35.400
1000×1000 pixels	36.023
1250×1250 pixels	33.974
Complete images	**25.849**

## Data Availability

The datasets and any remaining information can be obtained from the corresponding author upon reasonable request.
